# Biometric parameters and choroidal microstructure in Chinese children with unilateral anisometropia

**DOI:** 10.3389/fmed.2025.1576953

**Published:** 2025-07-31

**Authors:** Jing Wen, Yiwen Cao, Jingjing Zhao, Dehai Zhu

**Affiliations:** ^1^Department of Pediatric Ophthalmology, Peking University First Hospital, Beijing, China; ^2^Peking University Children Vision Institute, Peking University, Beijing, China

**Keywords:** unilateral anisometropia, axial length, choroidal thickness, choroidal vascularity index, EDI-OCT

## Abstract

**Purpose:**

This study aimed to investigate the biological parameters and choroidal microstructure of the eyes of children with unilateral anisometropia and to analyze the factors influencing refractive development in these patients.

**Methods:**

This observational cross-sectional study included 26 patients with unilateral hyperopic anisometropia and 34 patients with unilateral myopic anisometropia. Biometric parameters, including axial length (AL) and average keratometry (Ave-K), were measured. Choroidal thickness (CT) and vascular microstructure parameters, including the total choroidal area (TCA), luminal area (LA), stromal area (SA), and choroidal vascularity index (CVI), were obtained with enhanced depth imaging optical coherence tomography (EDI-OCT).

**Results:**

Compared to normal eyes, the AL and AL/K ratio were significantly lower in hyperopic eyes but significantly higher in myopic eyes (all *p* <0.001). The TCA and LA were significantly greater in hyperopic eyes and significantly lower in myopic eyes compared to normal eyes (all *p* <0.005). The LA/SA ratio and CVI were significantly lower in myopic eyes (*p* = 0.002, *p* <0.001) and non-significantly greater in hyperopic eyes than in normal eyes (*p* = 0.072, *p* = 0.050). In hyperopic anisometropia, interocular differences in both the AL and AL/K ratio were negatively correlated with subfoveal choroidal thickness (AL: *r* = −0.529, *p* = 0.005; AL/K: *r* = −0.612, *p* = 0.001) and the LA (AL: *r* = −0.393, *p* = 0.047; AL/K: *r* = −0.407, *p* = 0.039).

**Conclusion:**

Significant negative correlations between axial length and choroidal parameters (subfoveal choroidal thickness [SFCT]/ and luminal area [LA]) in hyperopic anisometropia suggest that axial elongation may be associated with choroidal thinning. The CVI decreases as hyperopia progresses to emmetropia and then to myopia, indicating that choroidal vascularity changes during refractive development.

## 1 Introduction

Anisometropia is defined as a difference in refractive error between the eyes that is greater than or equal to 1 diopter (D). Current estimates suggest that its prevalence among school-aged children ranges from 2 to 5% (1). Children with myopic anisometropia may not wear spectacles due to fusion difficulties, especially when the contralateral eye is emmetropic. Additionally, they may not receive refractive correction in the affected eye because they fail to perceive a decrease in visual acuity. Hyperopic anisometropia may contribute to amblyopia (2). Even if amblyopia does not develop, anisometropia can still cause problems (3, 4), such as visual fatigue, abnormal binocular vision, or stereopsis, due to the difference in the perceived size of images between eyes. Anisometropia represents a unique form of eye development, since different eyes of the same individual can develop different refractive states. Studies on patients with unilateral anisometropia can better explore the development of myopia, as these studies eliminate the influence of individual differences.

Recently, several studies have examined the various structural elements of myopic anisometropic eyes. A recent study of children with anisometropia revealed that several biometric parameters, such as axial length (AL), vitreous chamber depth (VCD), anterior chamber depth (ACD), and simulated K reading values, were significantly greater in more myopic eyes than in non-myopic eyes (5). Previous studies of adult patients have indicated that interocular differences in refraction among individuals with anisometropia are due mainly to variations in the posterior segment of the eye (6, 7). Liu et al. (8) reported that reductions in choroidal thickness and choriocapillaris vascular density were observed in the myopic eyes of individuals with anisometropia. However, the results of the choroidal microstructural changes reported in these studies were inconsistent. One limitation was that few patients with monocular myopia were included in previous studies. Therefore, further investigations of the interocular differences in children with unilateral myopic anisometropia are needed.

Few studies have examined interocular differences in patients with hyperopic anisometropia and no amblyopia. Some studies have shown that more hyperopic eyes have shorter axial lengths in children with hyperopic anisometropia (9, 10), but differences between other anterior and posterior biometric parameters have not been examined. Zhang et al. (11) examined choroidal thickness but did not investigate changes in choroidal vascular structure. Our previous study revealed that the choroidal vascularity volume was significantly greater in eyes with short axial length than in eyes with long axial length among anisometropia patients (12). However, it remains unclear whether the interocular differences between myopic anisometropia and hyperopic anisometropia are consistent.

Therefore, this study aimed to investigate the biological parameters and choroidal microstructures of eyes with different refractive statuses among children with unilateral anisometropia and analyze the factors influencing refractive development.

## 2 Methods

### 2.1 Participants

This observational cross-sectional study was performed in accordance with the Declaration of Helsinki and was approved by the Ethics Committee of Peking University First Hospital (PKUFH 2021–159). Written informed consent was obtained from all participants’ parents. The participants were consecutively recruited from the Department of Pediatric Ophthalmology at our hospital from October 2021 to February 2023. The inclusion criteria were as follows: (1) age between 6 and 14 years, (2) unilateral hyperopic/myopic anisometropia, with interocular differences in the spherical equivalent refraction (SER) of at least 1.00 D, and (3) best-corrected logarithm of the minimum angle of resolution (logMAR) visual acuity of 0 (20/20) or better in each eye. Notably, all included participants had best corrected visual acuity (BCVA) of 20/20 in each eye, ensuring that we examined the effects of anisometropia in the absence of amblyopia. The exclusion criteria were as follows: (1) a history of ocular or systemic diseases, including congenital cataracts and glaucoma, hypertension, and diabetes; (2) a history of amblyopia treatment; (3) a history of intraocular or refractive surgery; (4) a history of neurological disease; or (5) other evidence of retinal pathology. The demographic data (e.g., age and sex) of all enrolled patients were collected for analysis.

### 2.2 Ocular biological parameters

All participants underwent comprehensive ophthalmic examinations, and the following data were collected: best-corrected visual acuity (BCVA), SER, slit lamp biomicroscopy, and intraocular pressure (IOP). Retinoscopy measurements were performed under cycloplegia. AL, average keratometry (Ave-K), central corneal thickness (CCT), and ACD were measured via AL-Scan (Nidek Co., Ltd., Gamagori, Japan). The AL/K ratio was calculated by dividing the AL by Ave-K. The participants were categorized on the basis of the SER.

### 2.3 Choroidal microstructure

**Figure 1:**
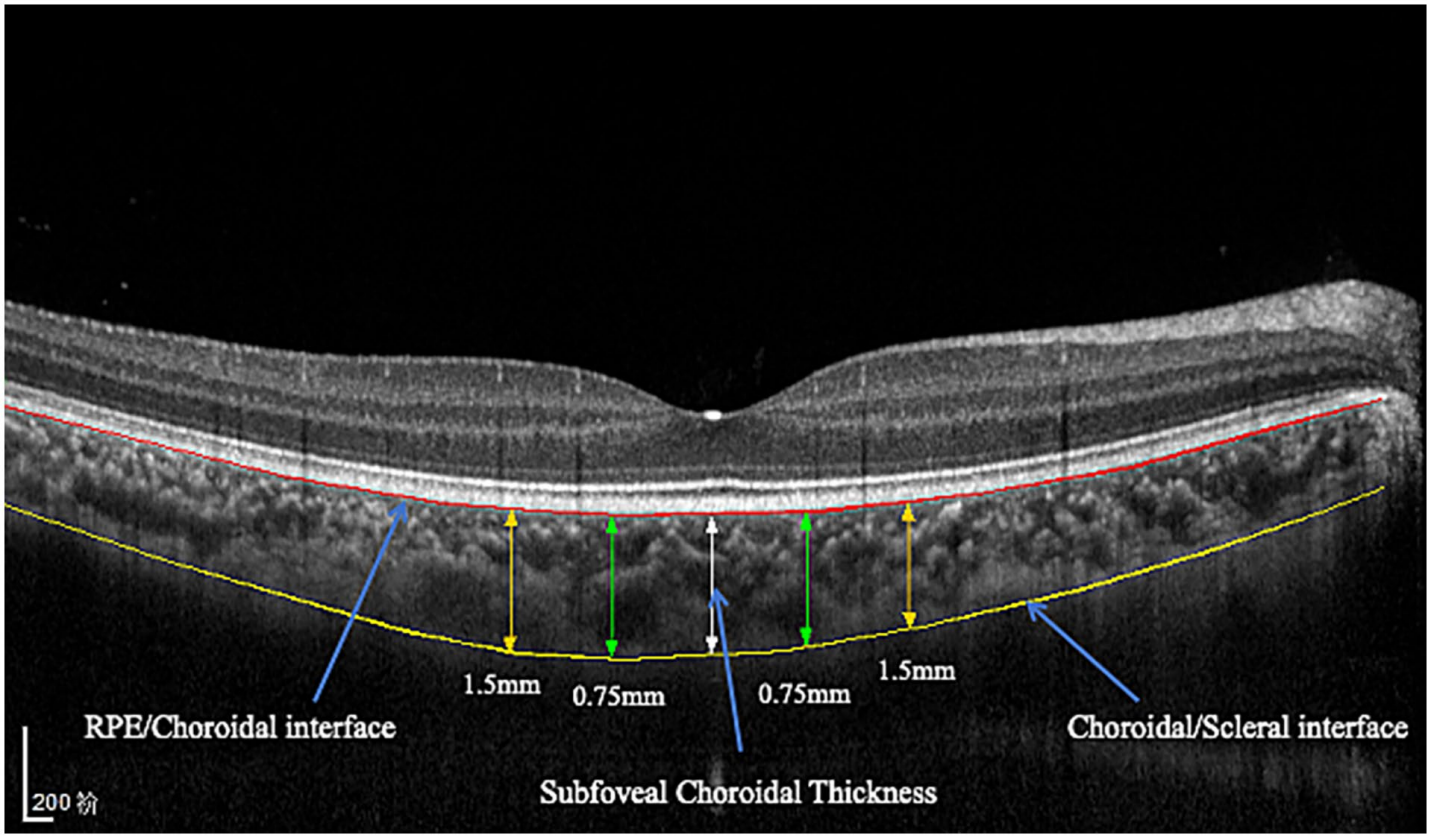
CT measurements on SD-OCT in EDI mode.

The choroidal area was imaged through spectral domain optical coherence tomography (SD-OCT, Spectralis, Heidelberg Engineering, Heidelberg, Germany) in the enhanced depth imaging (EDI) mode. All OCT images were obtained using the built-in eye-tracking system with 100-frame averaging to enhance the signal-to-noise ratio. Each image was required to have a signal strength of ≥30 (Heidelberg Spectralis quality scale, range 0–40) to ensure adequate image quality. All OCT examinations were performed at approximately 10 a.m. to reduce the influence of diurnal variations (13). The built-in AL adjustment function of the Heidelberg Spectralis OCT system was utilized (Heidelberg Engineering GmbH, Germany), which automatically selects from three predefined scanning modes (short, normal, and long AL) to optimize image scaling on the basis of individual biometric measurements. The choroidal thickness was measured from the outer border of the retinal pigment epithelium (RPE) and the inner surface of the hyperreflective line corresponding to the choroidal–scleral junction. Choroidal thickness measurements were obtained via both horizontal and vertical scans centered on the fovea. The measurements were taken at the fovea and at 0.75 mm and 1.5 mm nasally, temporally, inferiorly, and superiorly from the fovea (Figure 1). The subfoveal choroidal thickness (SFCT) was determined by calculating the average value of the SFCT on the horizontal and vertical meridians.

### 2.4 Image analysis

**Figure 2:**
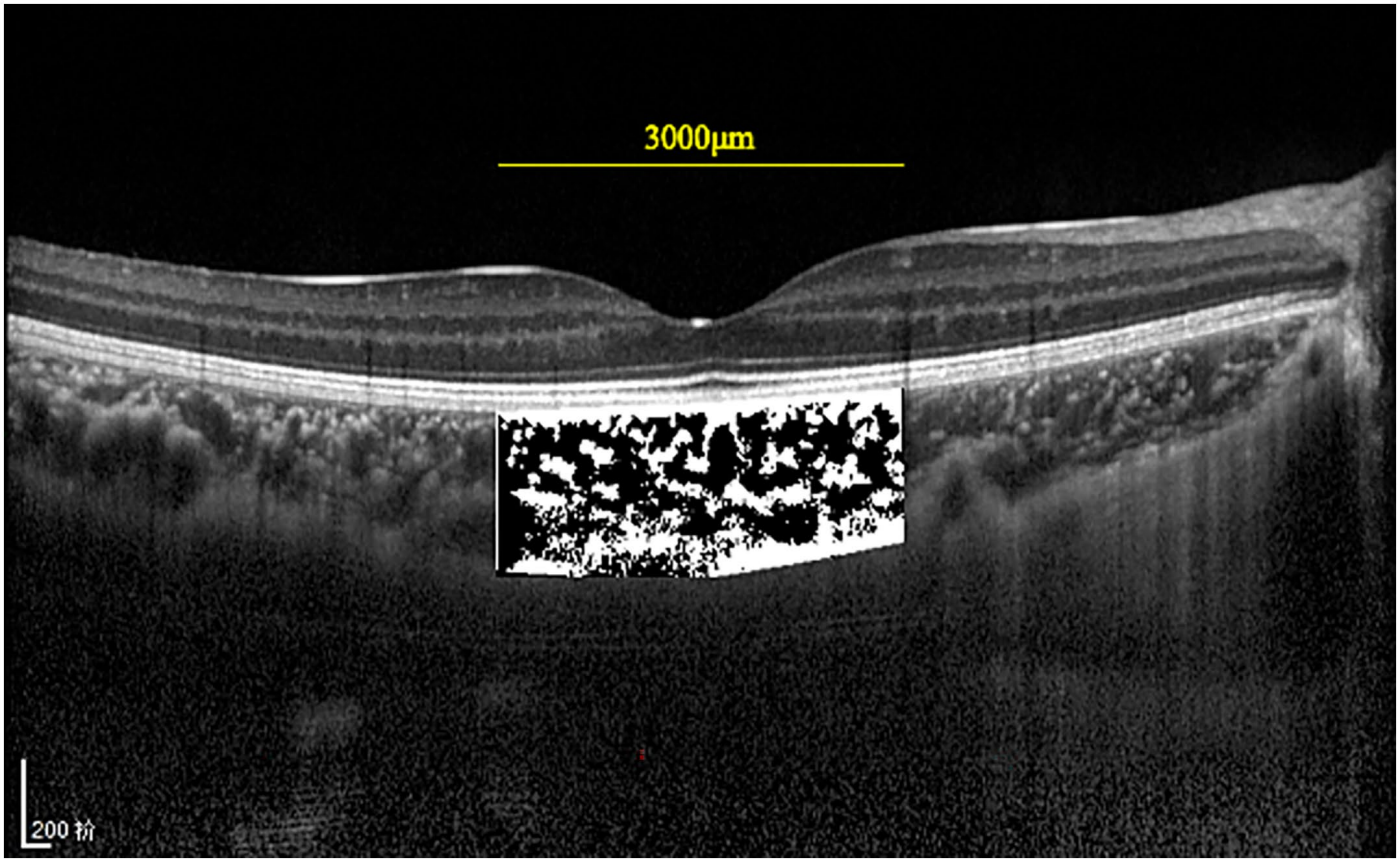
EDI-OCT images were binarized with dark and light pixels via Niblack. The luminal area (dark area) and the stromal area (light area) are 3 mm in size and are centered in the fovea.

ImageJ software (version 1.47, National Institutes of Health, Bethesda, MD, United States)^1^ was used for choroidal vascularity index (CVI) measurements (14). The examined area was 3,000 μm wide (1,500 μm on the nasal and temporal sides of the fovea) in the subfoveal choroid. The total choroidal area (TCA) was selected and measured. EDI-OCT B-scan images were binarized with dark and light pixels via the Niblack auto local threshold tool to determine the luminal area (LA) and stromal area (SA) of the choroid (Figure 2). The CVI was defined as the ratio of LA to TCA.

The OCT image magnification was adjusted using Bennett’s formula (15) to account for axial length (AL)-dependent scaling. The actual scan diameter (t) was calculated as t = p × q × s, where p is the device-specific magnification factor, q is the ocular magnification factor [q = 0.01306 × (AL − 1.82)], and s is the raw OCT measurement.

### 2.5 Statistical analysis

Data analysis was performed with Statistical Package for the Social Sciences (SPSS) version 23.0 (SPSS, Inc., Chicago, IL, United States). The distributions of the data were assessed for normality using the Shapiro–Wilk test. Normally distributed variables are presented as means and standard deviations, whereas non-normally distributed variables are expressed as medians and interquartile ranges (IQRs). Categorical data are presented as counts and percentages. For normally distributed data, group differences were analyzed using independent samples *t*-tests (for unpaired groups) or paired *t*-tests (matched groups). Non-normal data were analyzed with the Mann–Whitney U (independent) or Wilcoxon signed-rank tests (paired). Furthermore, the chi-squared test was used to compare sex ratios between the two anisometropia groups. Spearman’s rank correlation analysis was used to assess the associations between biometric parameters (i.e., SER, AL, and AL/K) and other choroidal parameters. A *p*-value of <0.05 indicated a statistically significant difference.

## 3 Results

### 3.1 General characteristics

A total of 60 subjects were enrolled in this study, including 26 patients with unilateral hyperopic anisometropia (female/male = 12/14, age 10.53 ± 2.51 years) and 34 patients with unilateral myopic anisometropia (female/male = 17/17, age 10.11 ± 1.72 years; Table 1). Eyes of the patients were categorized into four groups based on spherical equivalent refraction (SER): hyperopic eyes (SER ≥ +1.50D, group A), normal eyes in hyperopic anisometropia (−0.50D ≤ SER ≤ +1.00D, group B), normal eyes in myopic anisometropia (−0.50D ≤ SER ≤ +1.00D, group C), and myopic eyes (SER ≤ −1.00D, group D). The mean anisometropia value was 2.49 ± 0.61 (1.500–3.625) D in hyperopic anisometropia participants and 2.21 ± 0.98 (1.000–4.250) D in myopic anisometropia participants (*p* = 0.186, non-significant). There was no significant difference in the SER or AL between normal eyes of hyperopic and myopic anisometropia patients (*p* = 0.052 and *p* = 0.073, respectively).

**Table 1:** Demographic and clinical characteristics of the enrolled participants (*n* = 60).

Characteristics	*N*	Age (years)	Sex (male, %)	SER (D)	AL (mm)	Ave-K (D)	AL/K (mm/D)	CCT (mm)	ACD (mm)
Hyperopic anisometropia	26	10.53 ± 2.51	14 (53.85)						
Group A				2.63 [0.75]	22.24 ± 0.49	43.24 ± 0.89	0.51 ± 0.02	565.27 ± 28.59	3.59 ± 0.17
Group B				0.31 [0.56]	23.13 ± 0.50	43.44 ± 0.85	0.53 ± 0.02	563.35 ± 27.99	3.64 ± 0.17
*p*-value				<0.001	<0.001	<0.001	<0.001	0.072	0.005
Myopic anisometropia	34	10.11 ± 1.72	17 (50)						
Group C				0 [0.59]	23.42 ± 0.70	43.46 ± 1.43	0.54 ± 0.03	557.32 ± 27.51	3.74 ± 0.21
Group D				−1.88 [1.38]	24.26 ± 0.71	43.46 ± 1.46	0.56 ± 0.03	558.47 ± 27.76	3.79 ± 0.20
*p*-value				<0.001	<0.001	0.984	<0.001	0.398	<0.001
*p*-value		0.46^a^	0.77^b^						

ACD, anterior chamber depth; AL, axial length; Ave-K, average K-reading; CCT, central corneal thickness; SER, spherical equivalent refraction. The results are presented as the means ± SDs, medians [interquartile ranges (IQRs)], or *n* (%). ^a^Statistical significance tested with the independent-samples *t*-test. ^b^Statistical significance tested with the *χ*^2^ test. The Wilcoxon signed-rank test was used for interocular comparisons of SER. Paired-samples *t*-test was used for interocular comparisons of AL, Ave-K, AL/K, CCT, and ACD.

### 3.2 Ocular characteristics

Ocular characteristics, including AL, Ave-K, AL/K ratio, CCT, and ACD, are presented in Table 1. As expected, the AL of the hyperopic eyes (group A) was significantly shorter, and the AL of the myopic eyes (group D) was significantly longer (both *p* <0.001; Table 1). The AL/K ratio of hyperopic eyes was significantly lower than that of normal eyes, and the AL/K ratio of myopic eyes was significantly greater than that of normal eyes (both *p* <0.001). The Ave-K of the hyperopic eyes was lower than that of the normal eyes (p <0.001). Compared to normal eyes, myopic eyes presented a greater ACD, whereas hyperopic eyes presented a shallower ACD (both p <0.001).

### 3.3 Choroidal parameters measured by OCT

**Figure 3:**
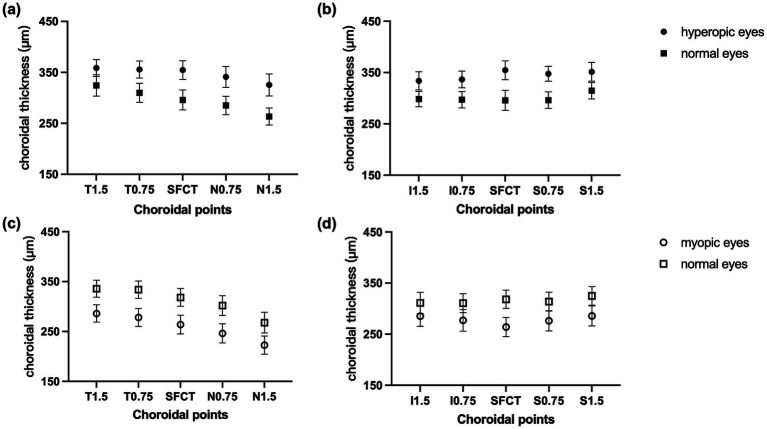
CT at different locations. CT of a horizontal B-scan at T1.5, T0.75, fovea, N0.75, and N1.5 in **(a)** hyperopic anisometropia and **(c)** myopic anisometropia patients. CT of a vertical B-scan at I1.5, I0.75, fovea, S0.75, and S1.5 in **(b)** hyperopic anisometropia and in **(d)** myopic anisometropia patients. The data are expressed as the means and 95% confidence intervals (CIs).

Table 2 shows that the SFCT was significantly greater in hyperopic eyes (group A vs. group B, *t* = 6.55, *p* <0.001) and significantly lower in myopic eyes (group C vs. group D, *t* = 8.70, *p* <0.001). The horizontal choroidal thickness in hyperopic eyes was significantly greater than that in normal eyes at 0.75 mm and 1.5 mm temporal and 0.75 mm and 1.5 mm nasal locations (all *p* <0.01; Figure 3a). The horizontal choroidal thickness in myopic eyes was significantly thinner than that in normal eyes at 0.75 mm and 1.5 mm temporal and 0.75 mm and 1.5 mm nasal locations (all *p* <0.001, Figure 3b). In the vertical direction, the choroidal thickness was significantly greater in hyperopic eyes at all points (all *p* <0.01; Figure 3c) and significantly thinner in myopic eyes at all points (all *p* ≤ 0.001; Figure 3d).

**Table 2:** Comparison of CT and choroidal vascular parameters (*n* = 60).

Parameters	Group A (*n* = 26)	Group B (*n* = 26)	*p*-value	Group C (*n* = 34)	Group D (*n* = 34)	*p*-value	*p*-value *(GA vs. GD)*
CT (μm, mean ± SD)							
SFCT	354.51 ± 45.46	295.89 ± 48.47	<0.001	318.18 ± 51.31	263.89 ± 53.97	<0.001	<0.001
N0.75	341.02 ± 50.75	285.09 ± 44.78	<0.001	301.86 ± 56.29	246.15 ± 54.77	<0.001	<0.001
N1.5	325.26 ± 53.41	263.46 ± 41.46	<0.001	267.73 ± 58.97	222.57 ± 52.23	<0.001	<0.001
T0.75	355.58 ± 41.33	309.80 ± 46.34	<0.001	333.79 ± 49.93	278.09 ± 51.57	<0.001	<0.001
T1.5	358.60 ± 41.17	324.34 ± 51.94	0.006	335.60 ± 48.44	285.93 ± 49.85	<0.001	<0.001
I0.75	336.58 ± 40.31	297.02 ± 39.28	<0.001	310.73 ± 53.02	277.08 ± 60.46	<0.001	<0.001
I1.5	333.79 ± 44.04	298.31 ± 37.23	0.001	311.00 ± 60.08	285.09 ± 56.25	0.001	<0.001
S0.75	347.64 ± 36.05	296.24 ± 39.72	<0.001	313.66 ± 52.84	276.13 ± 56.47	<0.001	<0.001
S1.5	351.46 ± 45.53	314.67 ± 39.60	0.002	324.84 ± 52.15	285.66 ± 56.29	<0.001	<0.001
Choroid vascular							
TCA, (mm^2^)	0.47 ± 0.06	0.43 ± 0.07	0.003	0.46 ± 0.07	0.41 ± 0.08	<0.001	0.002
LA, (mm^2^)	0.32 ± 0.05	0.29 ± 0.05	<0.001	0.31 ± 0.05	0.26 ± 0.05	<0.001	<0.001
SA, (mm^2^)	0.15 ± 0.03	0.14 ± 0.03	0.181	0.15 ± 0.03	0.14 ± 0.03	0.006	0.215
LA/SA	2.11 ± 0.37	2.00 ± 0.25	0.072	2.05 ± 0.35	1.83 ± 0.21	0.002	0.002
CVI (LA/TCA, %)	67.68 ± 3.72	66.35 ± 2.62	0.050	66.82 ± 3.28	64.51 ± 2.61	<0.001	0.001

CT, choroidal thickness; CVI, choroidal vascularity index; LA, luminal area; LA/SA, luminal area/stromal area ratio; SA, stromal area; SFCT, subfoveal choroidal thickness; TCA, total choroidal area; GA, Group A; GD, Group D; SD, standard deviation. N0.75, N1.5, T0.75, T1.5, I0.75, I1.5, S0.75, and S1.5 represent the choroidal thicknesses measured at 0.75 and 1.5 mm nasally, temporally, inferiorly, and superiorly, respectively, to the fovea. *P* value columns (for Group A vs. Group B, Group C vs. Group D) represent intra-group comparisons (paired-samples t-test) for each parameter. P value (GA vs. GD) column reflects the inter-group comparison (independent-samples t-test) between Group A (hyperopic anisometropia) and Group D (myopic anisometropia), as indicated by the footnote GA vs GD.

Significant differences were observed between hyperopic and myopic eyes in choroidal parameters, including CVI (*p* = 0.001), LA/SA ratio (*p* = 0.002), TCA (*p* = 0.002), and LA (*p* <0.001), while SA did not show a significant difference (*p* = 0.215). In children with hyperopic anisometropia, the TCA and LA in hyperopic eyes were significantly greater than those in normal eyes (*p* = 0.003 and p <0.001). The LA/SA ratio and CVI were slightly greater in hyperopic eyes than in normal eyes, but the difference was not significant (*p* = 0.072, *p* = 0.050; Table 2). In children with myopic anisometropia, the LA/SA ratio and CVI were significantly lower in myopic eyes (*p* = 0.002, *p* <0.001). The TCA and LA were also significantly lower in myopic eyes (*p* <0.001 for both), while SA showed a smaller but still significant difference (*p* = 0.006).

### 3.4 Relationships between choroidal parameters and ocular factors

The relationships between the choroidal parameters and the interocular differences in the SER, AL, and AL/K are presented in Table 3. In the hyperopic anisometropia group, the interocular difference in AL showed significant negative correlations with SFCT (*p* = 0.005) and LA (*p* = 0.047). In contrast, no significant correlations were observed in the myopic anisometropia group (see Supplementary Figure S1 for scatterplots). Structural patterns were visualized via EDI-OCT images (Supplementary Figure S2). Additionally, in the hyperopic group, the interocular difference in AL/K showed significant negative correlations with SFCT (*p* = 0.001) and LA (*p* = 0.039). In contrast, no significant correlations were found between SER, AL, or AL/K and any choroidal parameters in the myopic anisometropia group (all *p* > 0.05).

**Table 3:** Relationships between the interocular differences in the SFCT, TCA, LA, SA, and CVI and the interocular differences in the SER, AL, and AL/K ratio.

Parameters	SER	*p*-value	AL	*p*-value	AL/K	*p*-value
Hyperopic anisometropia						
SFCT	0.366	0.066	−0.529	0.005^*^	−0.612	0.001^*^
TCA	0.321	0.110	−0.366	0.066	−0.386	0.052
LA	0.357	0.074	−0.393	0.047^*^	−0.407	0.039^*^
SA	0.229	0.261	−0.329	0.101	−0.357	0.073
LA/SA	−0.081	0.693	0.182	0.373	0.244	0.230
CVI	−0.014	0.947	0.127	0.536	0.236	0.246
Myopic anisometropia						
SFCT	0.196	0.267	−0.253	0.149	−0.167	0.345
TCA	0.088	0.621	−0.242	0.168	−0.216	0.219
LA	0.077	0.664	−0.207	0.240	−0.193	0.273
SA	0.052	0.769	0.040	0.832	0.089	0.617
LA/SA	−0.025	0.888	−0.198	0.262	−0.250	0.153
CVI	−0.031	0.864	−0.189	0.284	−0.240	0.171

AL: axial length; CVI: choroidal vascularity index; LA: luminal area; SA: stromal area; SER: spherical equivalent refraction; SFCT: subfoveal choroidal thickness; TCA: total choroidal area. *Correlations were significant at a *p*-value of <0.05.

## 4 Discussion

Children with anisometropia constitute a useful population for studies on ocular structure in different refractive states because these studies explore differences between an individual’s two eyes. Our study investigated the biological parameters and choroidal microstructure of the eyes of children with unilateral anisometropia. We also analyzed the relationships between biological parameters and choroidal parameters. The results revealed significant differences in the AL and AL/K ratio between myopic and hyperopic anisometropia patients. Patients with myopia have greater ALs and AL/K ratios, whereas those with hyperopia have lower ALs and AL/K ratios. In patients with myopic anisometropia, the LA/SA ratio and CVI are lower in myopic eyes than in emmetropic eyes. In patients with hyperopic anisometropia, the LA/SA ratio and CVI are slightly greater in hyperopic eyes than in emmetropic eyes, but the differences are not statistically significant.

Numerous previous studies (8, 16–18) have shown that the development of myopia is accompanied by changes in choroidal vascularity, which are associated with increased AL. The LA/SA ratio and CVI are indicators of choroidal vascularity and provide information on the relative proportion of the choroid occupied by vascular and interstitial components. In children with myopia, the CVI (19) and choriocapillaris blood perfusion (20) are reduced. Similar results were observed in our study, with the LA/SA ratio and CVI significantly lower in myopic eyes than in normal eyes. These findings suggest that reduced choroidal vascularity may play a more significant role in the development of myopia compared to emmetropia in children.

Among the children with myopic anisometropia in this study, there was a significant reduction in the TCA and LA in myopic eyes compared to emmetropic eyes. This finding is consistent with those of multiple previous clinical studies (6, 7, 21), which described a continuous decrease in the TCA and LA in individuals with myopia. However, there may be differences in the rates of choroidal vasculopathy and matrix reduction at different stages of myopia development and progression. Several studies (6, 7, 19, 20) have reported a greater reduction in choroidal vascular components as the CVI and choroidal vascularity volume (CVV) decrease among patients with myopia. Some studies (22, 23) reported no change in the CVI among patients with myopia. Gupta et al. (24) reported a greater reduction in the SA than in the LA among patients with high myopia, resulting in a greater CVI among these individuals. The myopic anisometropic eyes in our study had low-to-moderate myopia, and the reduction in choroidal vascular components was obvious compared with that in the emmetropic eyes. These findings suggest that a decrease in the vascular luminal area and a change in the choroidal blood circulation may occur in the early stage of myopia development in children.

This study revealed an increase in the choroidal area and luminal area in hyperopic eyes. However, the difference in choroidal microstructure between hyperopic and emmetropic eyes varies greatly across studies. In a previous study of adults (25), emmetropic eyes had the highest CVI, whereas eyes with high hypermetropia and myopia had lower CVIs than emmetropic eyes. Chang et al. (26) reported that the TCA, LA, and SA were not significantly different between hyperopic and emmetropic eyes in children; however, choroidal vascular indices were not measured in this study. In previous studies on high hyperopia (27), the TCA, LA, and LA/SA ratios were significantly greater than those of normal eyes, while the CVI was lower. Our study involved children aged 6–14 years with monocular low-to-moderate hyperopic anisometropia, differing from the populations examined in the earlier studies. This inconsistency may be due to the differences in age, SE, or scanning patterns of the choroidal area across studies.

In the present study, the TCA and LA of hyperopic anisometropic eyes were higher than those of emmetropic eyes. Moreover, interocular differences in LA showed significant negative correlations with the differences in AL and AL/K ratio, suggesting that eyes with shorter axial lengths and lower AL/K ratios tended to have larger luminal areas. The findings of the present study align with those of a previous study on children with different refractive states (28), which showed that the CVI and LA/SA ratio were highest in hyperopia, followed by emmetropic eyes, and lowest in myopic eyes. This may be explained by the thickening of the choroid in hyperopic anisometropic eyes, which is primarily due to the increase in the area of the choroidal luminal area. Further research is needed to elucidate this topic.

Choroidal thickness (CT) is an important parameter for obtaining information about the choroidal layer. The present study revealed that the CT of hyperopic anisometropic eyes was greater than that of emmetropic eyes. In contrast, the CT of myopic anisometropic eyes was lower than that of emmetropic eyes, which is consistent with previous reports (8, 9, 29, 30). Jin et al. (31) reported that the central foveal CT was positively correlated with the SER and negatively correlated with the AL. Additionally, our study demonstrated a nasally to temporally decreasing trend in the difference in choroidal thickness (CT) in hyperopic anisometropic eyes, whereas no such trend was observed in myopic anisometropic eyes. This difference may be due to the inconsistency in the extent of changes in vascular and stromal components across different quadrants during CT changes. Although the thickness of the choroid is mainly determined by the luminal components, previous studies have reported that there are differences in the distributions of luminal and stromal components in different quadrants of the foveal and macular areas (32, 33).

Our study has the following limitations. First, we adopted a cross-sectional design. With the current sample size used in this study, which included 26 hyperopic and 34 myopic anisometropia participants, the power was 87% for detecting significant correlations (e.g., *r* = −0.529 for AL-SFCT in the hyperopic group). While our study was adequately powered to detect large effects (e.g., AL differences and strong correlations), the subgroup analysis may have been underpowered for subtle choroidal vascular changes. Specifically, the power to detect CVI differences in hyperopic anisometropia was only 46%, suggesting the possibility of type II errors in these analyses. Future longitudinal studies with larger populations are needed for more prospective and robust results. Another limitation is that we analyzed only the central 3-mm-wide subfoveal choroidal regions. The choroidal microstructure of a wide-field region outside this area might differ because eye growth is known to be asymmetrical. Finally, this study encountered technical limitations in choroidal imaging and binarization (34, 35). For example, images with low signal–to-noise ratios or measurement errors in manual segmentation may have led to decreased accuracy, which could have affected the reliability of the CVI as a reflection of choroidal vascularity. Future studies using a high-speed SS-OCT system (35) can improve choroidal vasculature imaging and choroidal angiography.

## 5 Conclusion

To our knowledge, this is the first study to focus on the differences in biometric parameters and choroidal vascular microstructure between unilateral hyperopic anisometropia and unilateral myopic anisometropia. Compared with normal eyes, myopic anisometropic eyes have greater axial lengths and AL/K ratios, whereas hyperopic anisometric eyes have lower axial lengths and AL/K ratios. We identified significant negative correlations between axial length and choroidal vascular parameters (SFCT and LA) in hyperopic anisometropia, suggesting a potential mechanistic link between axial elongation and choroidal thinning. The LA/SA ratio and CVI are highest in hyperopic eyes, followed by emmetropic eyes and myopic eyes. Choroidal blood flow may decrease as hyperopia progresses to emmetropia and then to myopia. In future studies, the association between refractive development and choroidal vascular structural changes (particularly CVI and luminal area variations) requires further longitudinal investigation using both OCT-based vascular indices and, where feasible, complementary time-resolved blood flow measurements such as OCT angiography (OCTA).

## Data Availability

The original contributions presented in the study are included in the article/Supplementary material, further inquiries can be directed to the corresponding author.

## Footnotes

^1^ http://imagej.nih.gov/ij/

## Glossary

AL: Axial length
Ave-K: Average keratometry
TCA: Total choroidal area
LA: Luminal area
SA: Stromal area
CVI: Choroidal vascularity index
EDI-OCT: Enhanced depth imaging optical coherence tomography
SFCT: Subfoveal choroidal thickness
D: Diopter
VCD: Vitreous chamber depth
ACD: Anterior chamber depth
SER: Spherical equivalent refraction
BCVA: Best corrected visual acuity
IOP: Intraocular pressure
CCT: Central corneal thickness
SD-OCT: Spectral domain optical coherence tomography
RPE: Retinal pigment epithelium
IQRs: Interquartile ranges
CVV: Choroidal vascularity volume
CT: Choroidal thickness
