# Supplementary material for: Biometric parameters and choroidal microstructure in Chinese children with unilateral anisometropia
Source: Front Med (Lausanne). 2025 Jul 31;12:1576953. doi: 10.3389/fmed.2025.1576953 (PMC12350249; doi:10.3389/fmed.2025.1576953)
Supplement: Supplementary file 1 [file Image_1.pdf]

(A) Interocular AL Difference vs. SFCT Difference

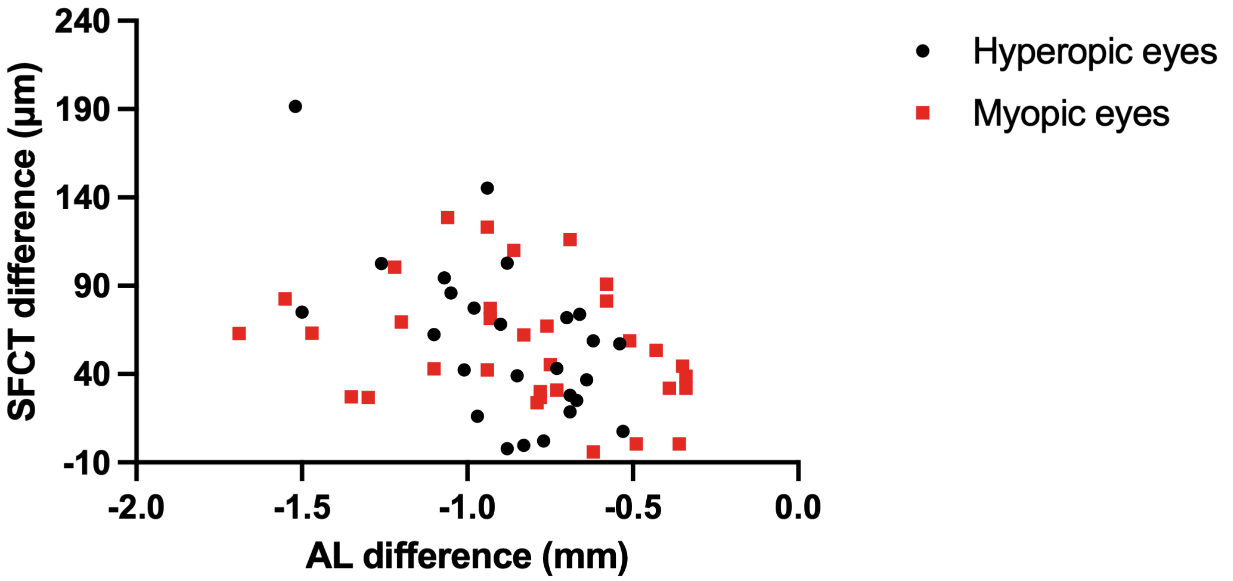

(B) Interocular AL Difference vs. LA Difference

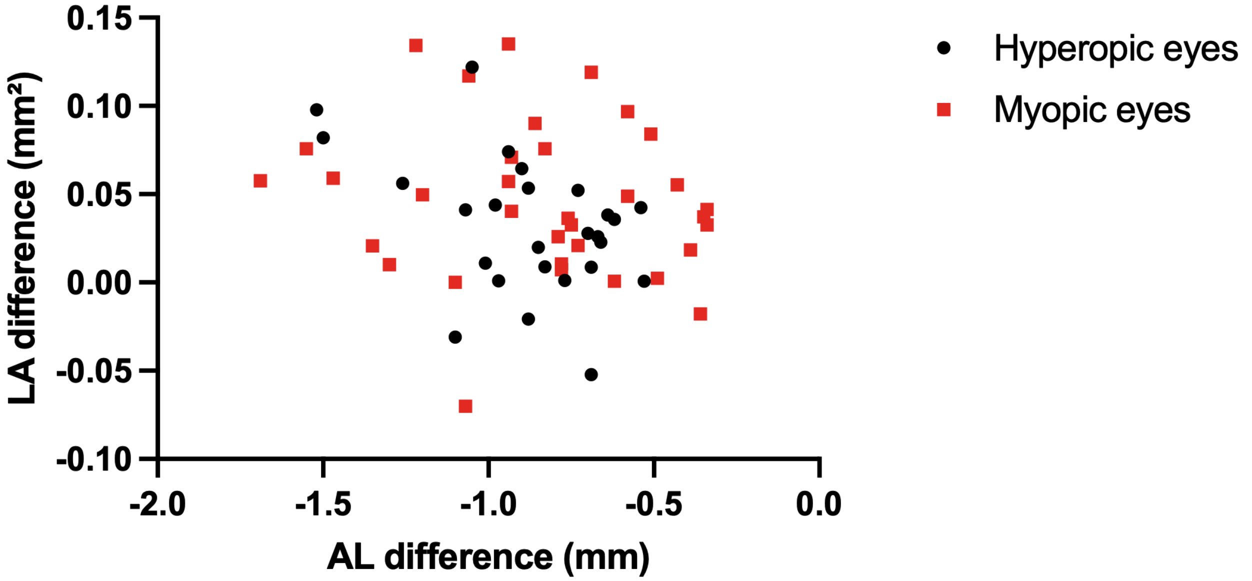

Supplementary Figure S1. Scatterplots of Interocular Differences in Biometric and Choroidal Parameters. (A) Correlation between axial length (AL) difference and subfoveal choroidal thickness (SFCT) difference. Hyperopic eyes (black) show a significant negative correlation ($r = -0.529$, $P = 0.005$), while myopic eyes (red) show no significant correlation ($r = -0.253$, $P = 0.149$). (B) Correlation between AL difference and luminal area (LA) difference. Hyperopic eyes show a significant negative correlation ($r = -0.393$, $P = 0.047$), while myopic eyes show no significant correlation ($r = -0.207$, $P = 0.240$). Data analyzed by Spearman's rank correlation.
