# Supplementary material for: Biometric parameters and choroidal microstructure in Chinese children with unilateral anisometropia
Source: Front Med (Lausanne). 2025 Jul 31;12:1576953. doi: 10.3389/fmed.2025.1576953 (PMC12350249; doi:10.3389/fmed.2025.1576953)
Supplement: Supplementary file 2 [file Image_2.pdf]

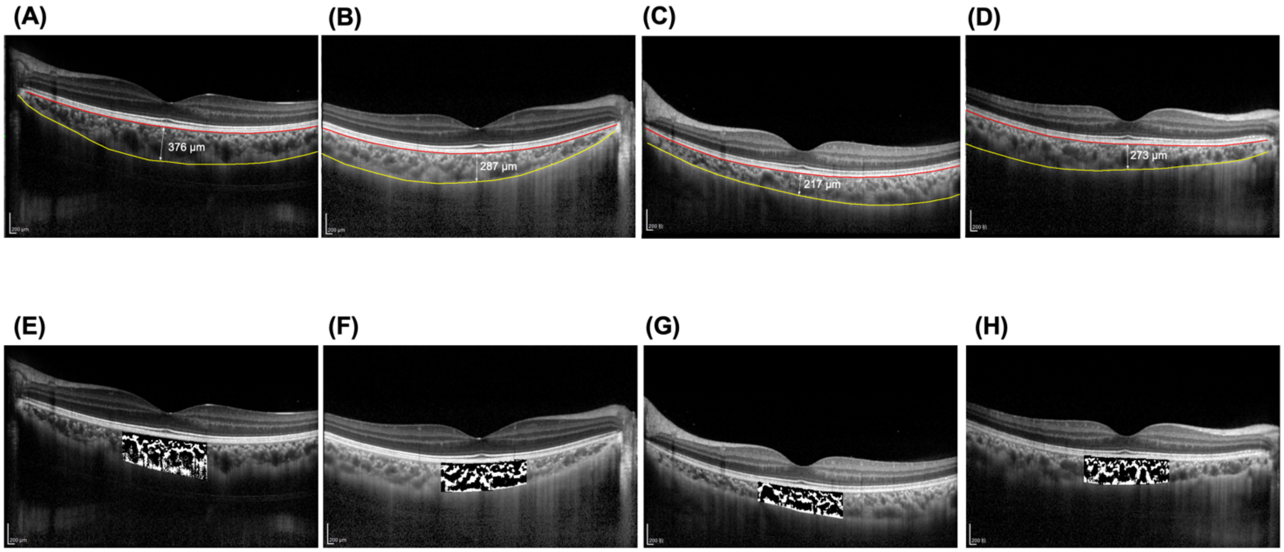

Supplementary Figure S2. Comparative EDI-OCT Images of Choroidal Structure. Hyperopic eye (A) shows thickened choroid with significant negative AL-SFCT correlation, compared to normal fellow eye (B). Myopic eye (C) exhibits thinner choroid without significant AL-SFCT correlation, versus normal fellow eye (D). Binarization was performed to quantify luminal area (dark pixels). Same hyperopic eye as (A) demonstrates enlarged LA (E) with significant AL-LA correlation, contrasting normal eye (F). Same myopic eye as (C) presents reduced LA (F) with nonsignificant AL-LA correlation, compared to normal eye (H).
